# Nonmedical Transdisciplinary Perspectives of Black and Racially and Ethnically Diverse Individuals About Antiracism Practices: A Qualitative Study

**DOI:** 10.1001/jamanetworkopen.2021.47835

**Published:** 2022-02-09

**Authors:** Megha Shankar, Joy Cox, Juliana Baratta, Gisselle De Leon, Jonathan G. Shaw, Sonoo Thadaney Israni, Donna M. Zulman, Cati G. Brown-Johnson

**Affiliations:** 1 Division of General Internal Medicine, Department of Medicine, University of California, San Diego; 2 Presence Center, Stanford University School of Medicine, Stanford, California; 3 Division of Primary Care and Population Health, Stanford University School of Medicine, Stanford, California; 4 VA Palo Alto Health Care System Center for Innovation to Implementation, Menlo Park, California

## Abstract

**Importance:**

Overwhelming evidence that anti-Black racism is associated with health inequities is driving clinician demand for antiracism practices that promote health equity.

**Objective:**

To investigate how nonmedical professionals address personally mediated, institutional, and internalized racism and to adapt these practices for the clinical setting.

**Design, Setting, and Participants:**

Using an approach from human-centered design for this qualitative study, virtual qualitative interviews were conducted among 40 professionals from nonmedical fields to investigate antiracism practices used outside of medicine. Inductive thematic analysis was conducted to identify latent themes and practices that may be adaptable to health care, subsequently using an established theoretical framework describing levels of racism to interpret and organize themes. Convenience and purposive sampling was used to recruit participants via email, social media, and electronic flyers.

**Main Outcomes and Measures:**

Antiracism practices adapted to medicine.

**Results:**

Among 40 professionals from nonmedical fields, most were younger than age 40 years (23 individuals [57.5%]) and there were 20 (50.0%) women; there were 25 Black or African American individuals (62.5%); 4 East Asian, Southeast Asian, or South Asian individuals (10.0%); 3 individuals with Hispanic, Latinx, or Spanish origin (7.5%); and 3 White individuals. Participants described personally mediated, institutional, and internalized antiracism practices that may be adaptable to promote health equity for Black patients. Personally mediated antiracism practices included dialogue and humble inquiry, building trust, and allyship and shared humanity; clinicians may be able to adopt these practices by focusing on patient successes, avoiding stigmatizing language in the electronic health record, and using specific phrases to address racism in the moment. Institutional antiracism practices included education, representation, and mentorship; in the health care setting, clinics may be able to develop staff affiliate groups, focus on improving racial health equity outcomes, and conduct antiracism trainings. Internalized antiracism practices centered on authenticity; clinicians may be able to write positionality statements reflecting their identity and the expertise they bring to clinical encounters.

**Conclusions and Relevance:**

This study’s findings suggest that antiracism practices from outside the health care sector may offer innovative strategies to promote health equity by addressing personally mediated, institutional, and internalized racism in clinical care.

## Key Points

**Question:**

What antiracism practices from transdisciplinary fields can be adapted to medicine to promote health equity for Black patients?

**Findings:**

In this qualitative study, 40 professionals from nonmedical fields described personally mediated, institutional, and internalized antiracism practices that may be applicable in health care.

**Meaning:**

These findings suggest that individual clinicians and clinics may be able to proactively incorporate antiracism practices into patient care to address personally mediated, institutional, and internalized racism experienced by Black patients.

## Teaser

This qualitative study collects perspectives from racially and ethnically diverse professionals in nonmedical fields on antiracism practices that may be applicable to health care.

## Introduction

Racism carries a long legacy in medicine and is recognized as a cause of illness.^1^ In the health care system, racial inequities in communication, a cornerstone of the patient-clinician relationship, are long standing; Black patients report experiencing lower-quality physician communication, less participatory decision-making, and shorter clinical visits.^2,3^ The American College of Physicians recommends systemic interventions paired with clinician adoption of antiracism communication practices to promote health equity.^4^

The seminal 2000 article by Jones^5^ published in the *American Journal of Public Health* described 3 levels of racism: personally mediated (prejudice and discrimination occurring between individuals), institutional (differing material conditions and access to power), and internalized (acceptance of negative messages by members of stigmatized racial and ethnic groups about their own abilities and intrinsic worth). The levels of racism framework can be used to examine health inequities and shape antiracism efforts. Although this framework emerged 20 years ago, only recently have organizations acknowledged institutional, or structural, racism (ie, racism occurring within institutions, systems, and structures of society) in health care.^6^

There is an opportunity for clinicians to learn antiracism strategies from other disciplines, adapting them to medicine. Borrowing approaches from nonmedical fields to improve the way health care is delivered is not new; one well-known example of this is the adoption of the Lean management system to health care quality improvement.^7^ Approaches derived from design thinking can focus on human-centered ways of problem solving and value interdisciplinary collaboration.^8^ Integrating analogous inspiration is an established approach in human-centered design to generate innovative solutions.^9^ This approach has successfully brought new viewpoints to existing challenges in medicine, such as patient-provider connection^10^ or insulin delivery.^11^ To address the multilayered issue of racism in medicine, it may be particularly helpful to learn from nonmedical disciplines, including those that are further along in antiracism efforts, and through an empathy-driven approach like human-centered design.^12^ There is an opportunity to bridge transdisciplinary antiracism communication practices to medicine through an approach that centers patients. We use the levels of racism framework to describe personally mediated, institutional, and internalized antiracism practices that may be applicable to medicine.

## Methods

For this qualitative interview study, we used a human-centered design approach that draws on the experiences of individuals in analogous professions to learn about transdisciplinary antiracism communication practices and how they may be applied to health care.^10^ Study activities were designated as exempt from human participants review by the Stanford University Institutional Review Board. Participants provided verbal informed consent to participate in this study and were informed that the findings may be published.

### Quality

The Consolidated Criteria for Reporting Qualitative Research (COREQ) reporting guideline checklist was completed to ensure quality of qualitative research was upheld. To address credibility, transferability, dependability, confirmability, and reflexivity, we embarked on a number of quality-building processes.^13^ We used race and ethnicity–concordant interviewers as often as possible to elicit the best-quality data from participants who may have been more comfortable sharing with race and ethnicity–concordant interviewers (credibility). All data received 3 rounds of coding to encourage correct interpretation. The expert physician team (including M.S., J.G.S., and D.M.Z.) that reviewed findings from our participants found the results applicable to health care (credibility and transferability). Future work includes additional focus groups and interviews with patients and providers that are in process and may further confirm or disconfirm stability of results over time and across populations.

### Reflexivity Statement

Our interdisciplinary and diverse research team provided a unique lens for this study. Among 10 interview team members, there were 3 Black or African American individuals; 3 East Asian, Southeast Asian, or South Asian individuals (30.0%); 2 individuals with Hispanic, Latinx, or Spanish origin (20.0%); and 2 White individuals. There were 9 (90.0%) women, and education ranged from undergraduate student (2 individuals) to doctoral degree (3 individuals) (eTable 1 in the Supplement). Interview guide development was shaped by team members’ qualitative and clinical medicine expertise, as well as lived experiences of team members who identified as members of racial or ethnic minority groups. When conducting interviews and qualitative analysis, our diverse lived experiences, including membership in gender and racial or ethnic minority groups and varied socioeconomic status (eTable 1 in the Supplement), informed probing questions and may have been associated with deeper responses from participants. The interview team considered the ways in which their own backgrounds, experiences, and assumptions may have been associated with their interactions with participants. We needed to address whether interviewers’ race and ethnicity and lived experiences may have been associated with participants’ willingness to talk openly about experiences with racism and antiracist practices or how this may have been associated with their responses. During data analysis, researchers (M.S., J.B., G.D., and C.G.B.J.) met weekly to discuss emerging codes and interpretation of key quotes. We discussed and challenged potential assumptions to develop a more holistic interpretation of the data.

### Participant Selection and Interview Process

We used convenience and purposive sampling to recruit 40 participants via email, social media, and electronic flyers. Participants had no prior connection or affiliation with the research team, except for personal contacts to authors M.S. and J.C. Among 43 individual recruitment emails and 3 social media contacts sent out, there were 36 initial responses. We also circulated a recruitment email via 1 listserv from which 17 individuals responded, and 13 individuals were lost to follow-up. The total number of contacted participants was 63, and 40 individuals were interviewed, so we had a 63.5% response rate. Potential participants outside of medicine were invited to interviews with the explicit goal of discussing “antiracist communication practices from a variety of occupations that can be applied to the patient-clinician relationship.” We monitored gender and age representation as our sample grew, prioritizing balanced gender and age representation, and we oversampled Black individuals and members of other racial and ethnic minority groups. Options for participant racial and ethnic responses were American Indian or Alaska Native; Black or African American; East Asian, South Asian, or Southeast Asian; Hispanic, Latinx, or Spanish origin; Middle Eastern or North African; Native Hawaiian or Other Pacific Islander; and White or European origin. Our final participant group’s professional roles represented many categories of the Standard Occupational Classification Manual^14^: management, business, computer, science, community service, legal, education, arts, and personal care. Participation was voluntary, and participants received a $25 gift card.

We developed a semistructured interview guide (eAppendix in the Supplement) using principles of qualitative interviewing, starting with appreciative inquiry.^15^ Open-ended questions focused on workplace experiences with anti-Black racism and specific antiracism communication practices. Because recounting racism experiences can be healing or retraumatizing, we piloted and adapted the guide iteratively to encourage open reflection and minimize retraumatization.^16^

An interdisciplinary research team (including M.S., J.B., and G.D.) (eTable 1 in the Supplement) trained and guided by 2 qualitative researchers (J.C. and C.G.B.J.) conducted individual audio-recorded interviews over Zoom from the interviewers’ homes. Interviews ranged from 35 to 73 minutes (mean, 46 minutes). To the interviewers’ knowledge, no other individuals were present during interviews. Interviewer training included a 1-hour didactic session led by qualitative research expert J.C. on best practices for qualitative interviewing. Novice interviewers observed 1 to 2 interviews first and conducted their first interviews in a pair with M.S. or J.B. Field notes were taken only postinterview. We used the Scribie professional transcription service to transcribe interviews. Thematic saturation was used to determine appropriate sample size. Interviews were conducted by a team with diverse racial and ethnic backgrounds and professional expertise. Among 27 interviews with Black or African American participants, 11 interviews (40.7%) were racially concordant.

### Data Analysis

First, we used an inductive thematic analysis approach to identify latent themes.^17^ Four coders (M.S., J.C., J.B., and G.D.) conducted first-round independent coding (2 transcripts), second-round nonindependent verification coding (3 transcripts), and third-round nonindependent double coding using Dedoose version 8.3.35 (Dedoose; 35 transcripts).^18^ We used 7 codes for this analysis: dialogue and humble inquiry, building trust, allyship and shared humanity, education, representation, mentorship, and authenticity (eTable 2 in the Supplement). Qualitative experts (J.C. and C.G.B.J.) confirmed the most salient themes to interpret and structure our initial findings, mapping these to Jones’ levels of racism framework^5^ antiracism categories: personally mediated, institutional, and internalized.

Finally, to adapt emergent antiracism recommendations from this sample to health care, our team (with 5 clinicians, including M.S., J.G.S., and D.M.Z.) relied on consensus analysis approaches; we iteratively developed and refined initial analogous health care practices in weekly research team meetings. Five clinicians who are also educators and researchers with formal experience and expertise in diversity, equity, and inclusion provided written feedback on the proposed adapted health care practices.

## Results

Table 1 shows participant demographics and occupational categories. Most interviewees identified as Black or African American, with remaining participants identifying as American Indian or Alaska Native; East Asian, Southeast Asian, or South Asian; Hispanic, Latinx, or Spanish origin; or White or selecting multiple racial or ethnic identities. Interviewees’ ages ranged from 18 years to 60 years or older, with balanced gender representation. Among 40 professionals from nonmedical fields, most were younger than age 40 years (23 individuals [57.5%]); there were 20 (50.0%) women, 19 (47.5%) men, and 1 nonbinary individual (2.5%). The study population included 1 American Indian or Alaska Native individual; 25 Black or African American individuals (62.5%); 4 East Asian, Southeast Asian, or South Asian individuals (10.0%); 3 individuals with Hispanic, Latinx, or Spanish origin (7.5%); 3 White individuals; and 4 individuals with multiple race or ethnicity selections. Study participants came from the following occupation categories: management, education instruction and library, legal, life, physical, and social science, community and social service, personal care and service, and arts, design, entertainment, sports, and media.

**Table 1.** Interview Participant Demographics

Characteristic	Participants (N = 40)
Occupation	
Management	5 (12.5)
Education instruction and library	10 (25.0)
Legal	3 (7.5)
Life, physical, and social science	6 (15.0)
Community and social service	8 (20.0)
Personal care and service	6 (15.0)
Arts, design, entertainment, sports, media	2 (5.0)
Race and ethnicity^a^	
American Indian or Alaska Native	1 (2.5)
Black or African American	25 (62.5)
East Asian, Southeast Asian, or South Asian	3 (7.5)
Hispanic, Latinx, or Spanish origin	3 (7.5)
White or European origin	4 (10.0)
Multiple selections	
Age, y	13 (32.5)
18-29	10 (25.0)
30-39	8 (20.0)
40-49	4 (10.0)
50-59	2 (5.0)
≥60	3 (7.5)
Missing	
Gender	20 (50.0)
Women	19 (47.5)
Men	1 (2.5)
Nonbinary	5 (12.5)

^a^ Participants were asked to select all racial and ethnic identities that applied. Options for participant racial and ethnic responses were American Indian or Alaska Native; Black or African American; East Asian, Southeast Asian, or South Asian; Hispanic, Latinx, or Spanish origin; Middle Eastern or North African; Native Hawaiian or Other Pacific Islander; and White or European origin.

Participants discussed personally mediated, institutional, and internalized antiracism practices from Jones’ levels of racism framework^5^ (eFigure in the Supplement). Table 2 shows representative excerpts for 7 codes mapped to the 3 themes: dialogue and humble inquiry, building trust, allyship and shared humanity (personally mediated antiracism); education, representation, mentorship (institutional antiracism); and authenticity (internalized antiracism). Table 3 shows applications of antiracism practices to health care.^19,20,21,22,23,24,25,26,27,28,29,30,31,32^

**Table 2.** Excerpts From Transdisciplinary Qualitative Interviews on Combatting Anti-Black Racism

Theme	Code	Excerpts
Personally mediated antiracism	Dialogue and humble inquiry	“In the instance of racism I experienced, I literally was so stunned that I had no knowledge of what to do … I think it'd be good to have almost like a plan of action, like if you had a script or like, ‘Hey, this weird thing has happened and here's like, I'm ready.’ Then it's like if that's in there, then it's possible. You'll be like, ‘Oh, here's a few things that I can say to maybe address it.’”
		“Dialogue is so important, and the idea is that you first start out with understanding your stakeholders, and you do that by crafting an empathy map, that you have to go through the hard work of understanding who you're designing some kind of a resolution for. And that involves conversations, that involves interviews, that involves outreach. It involves being incredibly vulnerable, stepping outside of your own perspective to understand others. It involves intentional listening.”
		“There are a lot of discussions, and questions that people bring up and talk about have been, ‘How does white supremacy impact you specifically? How do you feel it affects the way you're allowed to express yourself?’ Thinking about ways in which we may have internalized white supremacy, what are capitalist or market-based structures that imposed white supremacy on all of us? And then depending on the audience things can get a little more specific, like I have been in one where there are a lot of people who were either new parents or expecting parents, and so there was a lot of discussion on how do you raise antiracist children? How do you raise antiracist Brown children? How do you raise antiracist White children? And so, yeah, I think they tend to start pretty general and then can go in different directions based on what people are sharing.”
	Building trust	“So now, it's not just that, oh, there's some random Black person hundreds of miles away and got killed by the cops, but it's like, I interact every day with a person of color, who I love and who I trust in who I see as like a role model and a mother figure. So, it brings light to them and these are things that aren't OK.”
		“When I have reached out to clients, there's an easing up that they often feel where they're like, ‘This is someone who maybe I could trust a little bit.’ And I try to use that as a potential bridge. …. And there's a lot of things that can be points of potential division, but there is an opportunity, I think, for Black and Brown people to really see a connection that, I think, is really important. That's meant a lot to me, and it doesn't in any way mean that you can't make important comparable connections with clients or other folks that you're working with if you're White or not a person of color. It's just that it's a different set of opportunities and strategies that occur.”
	Allyship and shared humanity	“Being an ally is to be in solidarity and to give space to the pain and the frustration that that community is feeling. And to also be intentional listeners of folks who need to share, whatever that means, those experiences, and to give voice and uplift those voices in any forums where we can. So I draw on my own peacemaking background just to embrace intentional listening of those and to be in spaces where I can understand and hear and support my Black colleagues … any way I can.”
		“If I see something happening to a Black person, I'll speak up. I think it's also easier for me to stick up for other people than it is for myself. So I will always address it or say something. If I don't feel safe enough to do that or if I don't feel like it's the time, I will reach out to the Black person and like express my support or ask what they need, asking how I can support them. I think if it's something I've experienced, I might be like, ‘I really empathize with this or resonate.’ I think I tried to stay away from saying, ‘I understand,’ if I don't. Like sometimes you think you might understand, but you don't actually, and I think that's something from allies that I would like to see more. I think saying you understand when we know that you don't quite understand what it's like to experience anti-Blackness is just not helpful. So expressing support, asking how you can support someone is really important.”
Institutional antiracism	Education	“Antiracism requires really intense study … we had a book series where the first book was the role of education for Black people in the South, from the Civil War to World War II. The second book we read was *The Warmth of Other Suns*. We read then a really difficult book on slavery, the history of convict leasing and the penal system … we're reading now *The Color of Law*, which is like the history of the government in the banking industry and all of that, you know, deliberately segregating people throughout the United States … the whole reasoning is to really educate people about systemic racism so that we can be informed … seeing some of the laws are written in ways that appear to be race neutral but that clearly once you learn are not.”
		“Our organization has a specialized training called mind sciences. It addresses implicit bias and microaggressions in the workplace. A lot of trainings deal with undoing history, undoing the white washing of history, because you still hear a lot of anti-Blackness. It takes having those real, you know, facts like saying, ‘Hey, this is what happened, this is how this came about.’ And that's hard to do in the workplace. We're gonna take everyone over to do a whole training for a year, rewire brains to not be racist. I think that goes back down to structural things in our country, like the education system, have it be antiracist because it's not antiracist right now. It's barely even inclusive. Having one month of Black history is not inclusive. Trying to do that on the level where you have adults that have been trained and taught their entire lives to think one way. And you're trying to undo that just in the workplace.”
	Representation	“We all need to see ourselves. We need to see people who look like us presenting or writing or in these leadership positions in a way that is meaningful and not tokenism.”
		“Improving representation involves specific recommendations about recruitment, outreach, hiring, who's on a search committee, what kind of questions you ask on a search committee. What are the trainings that search communities need to have to sort of reflect their own potential biases? How do we create very intentional pathways for professional development? How do we change the campus climate? We made some specific recommendations for that. A lot of that conversation impacted many of our communities of color, but the most poignant were our Black staff in their comments. And so that's one way I've been able to start to rethink and to help educate all my colleagues and myself as well in this process and move through a process that can hopefully make some change. We are moving toward creating toolkits for processes, for hiring and advancement and recruitment and training. That's one example of how I've been able to take my concerns about anti-Black racism and translate them into a staff cohort.”
	Mentorship	“With my mentor-mentee relations, [individuals] tell me about something that happened that hurt them and was anti-Black toward them or felt anti-Black, though it was something that wasn't overt racism. And I'll let them know that it is what it was. However, they felt it was, it is that. It was that. What do you need and … Or, yes, that happened, and I'm sorry about that. I'm gonna use my connection to Whiteness also as my privilege. I am very much connected to Whiteness, and I have that privilege, and so I can put myself in front of that situation too, however that looked subtle or overt, I will do it because I have that connection. And though it might come back on at me, it'll come back at me different than it might come back at my African American mentee, maybe they have a little bit of peace of mind or they have to fight a little less, because I know, I hope that people do that for me too.”
		“There have been a ton of affiliate groups that have been set up within our organization for different types of groups, and they range from Black men's affinity groups to people with disabilities and people who are caregivers. I think these have been valuable 'cause it provides people with a space where, I think, they have mentorship from others with shared understanding and they can be seen and talk about things.”
Internalized antiracism	Authenticity	“The first thing that I do is I announce my Blackness and its authenticity, because I think that that's important to allow people to understand that this is a safe space to be Black. And as you want to be without judgment of you know, not using the King's English or not knowing something or not having read Shakespeare or even reading the latest books that are on the top 10 on The New York Times. And this is not just for Black people, but I always do that especially if there are other Black people in the space to ensure that they feel safe enough to be the same person that they would be if we were family. Or if this was a space just for Black people, because I think all people of color but Black people, more specifically, we have we've become used to compartmentalizing just how much of ourselves, we bring to a space, whether it be digital or physical.”
		“Part of the work is being authentic, being self-aware. I think part of doing this external work is doing a lot of internal work, and being aware of who you are, what your privileges are. And that's the first step, and I think it's the hardest one. And I engaged in it very deeply when I wrote my dissertation, and it was emotional, it was hard, it was a long process, but I do think it's really important to know all of those things about yourself, and especially what privilege you do have in this world. As well as what you don't. I think both are important, but those certainly will shape the way that you see people and in turn, the way they see you and feel comfortable being their authentic selves too, not having to hide anything.”

**Table 3.** Transdisciplinary Antiracism Practices and Adaptation to Health Care

Theme	Health care practice summary^a^
Personally mediated antiracism	
Intentionally use positive language to promote self-confidence	Celebrate patient health successes. Use positive language in the [electronic medical record] EMR (replace stigmatizing language such as “Patient is noncompliant with medications” with specific language such as “Patient is working hard on health and unable to pay for medications”). Other common negative language to avoid includes “poor historian” and “difficult patient.”^19^
Use prepared phrases to address antiracism in the moment	Use prepared phrases for addressing racism in clinical encounters (eg, “I'm having a really hard time with what you said. Can you tell me why you said that?” or “I found that very offensive. I'm sure it wasn't your intention.”)^20^
Talk directly to members of the community	Create a Black patient advisory board with regular meetings to learn about Black community priorities in health care^21^
Display signage aligned with racial justice to promote a workplace culture of inclusivity	Wear a white coat pin and display signage in the clinic with language that aligns with racial justice^22^
Conduct mental health check-ins	Using a collaborative care model, conduct mental health screenings and culturally sensitive treatment with Black patients regarding mental health effects of racism^23^
Offer tangible time for your Black colleagues	Consider health effects of racism when providing resources and support for time off work and disability assessment^24^
Institutional antiracism	
Provide intentional advocacy and mentorship to Black clients	Advocate and mentor on patient rights and when receiving health care (eg, coach patients to ask for a chaperone during sensitive exams). Intentionally use “warm handoffs” when referring to specialty providers to advocate for patients.^25^
Develop affiliate groups	Develop affiliate-interest groups for clinic staff to discuss issues and brainstorm solutions around racism in clinical care^26^
Hire individuals for diversity, equity, and inclusion efforts at the workplace	Hire administrative-clinical manager or appoint an existing staff member as a lead to focus on clinic-wide racial health equity efforts and metrics^27^
Conduct educational trainings on anti-Black racism specifically, rather than racism broadly	Conduct required clinical trainings, providing dedicated continuing medical education (CME) time, on the history of anti-Black racism in medicine^28,29,30^
Don’t call the police if deescalation is an option	Implement clinic policy to be thoughtful about when to involve the police, security, or other health care–related regulatory bodies to address patient issues. Provide deescalation training to all patient-facing staff.^31^
Internalized antiracism	
Write a positionality statement	Include a positionality statement as a part of online medical professional profile describing aspects of identity, values, and expertise a clinician brings to clinical care^32^

^a^ Examples from health care are cited throughout.

### Personally Mediated Antiracism

Participants discussed 3 major themes relevant to personally mediated racism (ie, combatting prejudice and discrimination occurring between individuals). These were dialogue and humble inquiry, building trust, and allyship and shared humanity.

#### Dialogue and Humble Inquiry

Participants most frequently identified dialogue and humble inquiry as the starting point for personally mediated antiracism practices. Participants noted the importance of engaging in dialogue around microaggressions, bias, and racism and embracing the discomfort that may accompany these conversations. Participants described productive dialogue as coming from a place of curiosity, occurring among individuals from diverse backgrounds, happening in public settings and occurring one on one, and taking place regularly and over time. One participant suggested spending 5 minutes per day with an individual to intentionally get to know the individual better, making sure to use positive language to avoid deficit thinking.^33^ Another participant used progressive stack,^34^ an approach in which facilitators cue people to speak in meetings to ensure voices are heard equitably. Participants recommended specific language to use when engaging in conversations around racism to demonstrate a commitment to growth and personally mediated antiracism.

#### Building Trust

Participants described building trust as an antiracism practice critical for meaningful interaction and founded on a belief in acting in another’s best interest with honesty and competence.^35^ Several participants, including educators, community organizers, and lawyers, described consistently “showing up” to campaigns and townhalls and actively listening as the foundation of building trust. Participants regarded rebuilding trust as equally important, given widespread historical instances of workplace structural and interpersonal racism. For example, a participant used empathetic listening, validating concerns, and displaying understanding to repair a relationship with a Black client who had experienced harm from other professionals; another participant held herself accountable through a formal apology to rebuild trust with Black employees.

#### Allyship and Shared Humanity

Participants recommended demonstrating allyship through acknowledging shared humanity as an antiracism practice. Black participants emphasized the importance of individuals of other races and ethnicities using their privilege in society to speak up in moments of racism to center Black voices and in instances when Black individuals may not have the opportunity to speak themselves owing to lack of power or fatigue. One participant gave a concrete example of displaying allyship for a Black colleague:

We had a client meeting at the same time our company had a scheduled memorial for George Floyd. I told my Black colleague, “I'll take the meeting, you should go attend the memorial.” I was able to support my colleague and tell my boss, “She's going to be at the memorial,” so that she didn't have to feel pressure to ask our manager herself.

Participants described acts of allyship as stemming from recognizing shared humanity. This includes practicing empathetic listening and intentionally taking the time to understand lived experiences of Black individuals. For example, a participant asked people about a time they felt different to explain what it might feel like to be from a marginalized community. One participant described the importance, as a step toward antiracism, of taking the time to get to know Black people in a way that is “intentional, thoughtful, tenacious, and loving” and understanding their lived experience. Members of other racial and ethnic minority groups who were not Black acknowledged shared humanity through cross–racial and ethnic solidarity, defined by one participant as working together with the understanding that struggles of diverse minority racial and ethnic communities are interconnected.

#### Practices for Personally Mediated Antiracism in Health Care

To engage in antiracism dialogue, clinicians may be able to learn prepared phrases that communicate humble inquiry and address racism in clinical encounters. In medical communication and documentation (eg, in discussion with colleagues, referrals, and visit notes), clinicians may be able to positively reframe deficit thinking and stigmatizing language with recommended alternatives (Table 2). To build trust and address health outcomes associated with racism, clinicians may be able to show up for patients, allowing patients to direct conversations about their treatment plans, ensuring they know the social, community, and health resources available to them. To demonstrate allyship, clinicians may be able to support patients taking time off work (eg, providing a doctor’s note) owing to health outcomes associated with racism.

### Institutional Antiracism

In discussing practices aimed at institutional racism (ie, differing conditions and access to power), 3 themes arose in promoting racial equity. These were education, representation, and mentorship.

#### Education

Participants repeatedly emphasized frequent and ongoing education as a crucial practice in combating racism, including learning the history of racism, antibias training coupled with reflection, and online spaces to share antiracism resources. Several White participants noted their lack of accurate education around racism and the importance of learning about systemic racism. One participant cautioned against conflating Black lived experience with diversity: “Here, we get ‘Diversity, Equity, and Inclusion,’ which are different than focusing specifically on anti-Black racism … it keeps us from getting to the real specifics of anti-Black racism and how anti-Black racism shows up in the lives of” individuals. Another participant highlighted related trainings, such as deescalation training to avoid involving the police or other regulatory bodies when not necessary, especially given the historical context of racial profiling. Participants repeatedly emphasized having structured education as a cornerstone to antiracism in their workplace.

#### Representation

Participants highlighted representation as paramount in combating institutional anti-Black racism. They described targeted programs for recruitment, hiring, and retaining Black individuals in the workplace at all levels. Participants noted the importance of representation and positive role modeling. Additionally, participants described the importance of Black individuals being represented in leading compensated workplace antiracism efforts, balanced by an equitable distribution of work burden across diverse groups.

#### Mentorship

Participants discussed formal mentorship (eg, a Black men’s affinity group) as an antiracism practice. Black participants described mentoring each other on structural workplace issues around perception, gender, power, and communication. A participant emphasized the importance of affinity groups for individuals who are not Black to brainstorm ways to provide intentional mentorship to Black individuals while reflecting on the complexities of workplace racism.

#### Practices for Institutional Antiracism in Health Care

Institutional antiracism in health care typically requires a change in clinic structure, process, or policy; participants suggested that the reason for this required change was that structures, process, and policies were historically focused on White populations. For education, clinics may be able to require yearly antiracism trainings, for example, on the national and local history of anti-Black racism in medicine, as a part of continuing medical education to promote true understanding of the Black narrative in health care. Clinics may be able to promote hiring practices that prioritize Black representation. Supporting peer mentorship, clinics may be able to develop work groups (including racially and ethnically concordant affiliate groups as possible) for staff to discuss issues and brainstorm solutions around racism in clinical care.

### Internalized Antiracism

Interviewees consistently identified authenticity as a key practice in addressing internalized racism. This consists of working to counter individuals’ acceptance of negative messages, stigma, and doubts of intrinsic worth.

#### Authenticity

Participants described practicing authenticity to address internalized racism, which involves believing in the components of assimilation into Whiteness and devaluing the stigmatized group.^36^ To address this, one participant described how he brings his authentic self to interactions: “I always announce my Blackness … to allow people to understand that this is a safe space to be Black … to ensure that they feel safe enough to be the same person that they would be if we were family. … We have become used to compartmentalizing how much of ourselves we bring to a space.”

Participants described authenticity as manifesting through first recognizing one’s true self and then taking the courageous steps of showing one’s true self in professional spaces. Black participants also encouraged physical expressions of authenticity, such as wearing their hair naturally or speaking with a nondominant accent, regardless of professionalism perceptions. Participants from racial and ethnic minority groups who were not Black described internalized racism experiences associated with imposter syndrome and the need to overcompensate by behaving in ways that conform to dominant White culture; being authentic was an important component of antiracism to combat imposter syndrome associated with internalized racism and disrupting the normative nature of Whiteness. One individual discussed how she portrays her personality through her dress and language, which she stated allows her to better connect with others. Another individual reported writing a positionality statement prior to conducting research to express her authentic self and reflect on her identity.

#### Practices for Internalized Antiracism in Health Care

When clinicians are comfortable expressing their authenticity and disclosing aspects of their identity, patients may feel invited to also bring their authentic selves to the clinical encounter, regardless of clinician and patient race and ethnicity concordance. Clinicians may choose to dress in a way that authentically aligns with their personal and professional identities, and clinics and organizations may be able to encourage this to destigmatize diverse cultural expressions of professionalism. Clinicians may be able to write positionality statements for their online clinical profiles or introduction videos, describing with humility their identity and the perspectives they bring to patient care, acknowledging their role as a guest in their patients’ lives.

## Discussion

By using human-centered design to explore antiracism practices in nonmedical disciplines in this qualitative study, we identified transdisciplinary strategies that may be applicable to health care and associated with increased trust, racial healing, and transformation.^37^ We found specific practices to address Jones’ personally mediated, institutional, and internalized levels of racism.^5^

In speaking with individuals from diverse occupations, our study builds on prior qualitative work on perspectives from patients and health care providers on racism in medicine, and we learned about essential antiracism communication practices not yet widespread in medicine. Prior research documents that clinician racial bias is associated with negative outcomes in the language used in a clinical encounter.^35^ Our findings suggest that clinicians may have an opportunity to intentionally address personally mediated racism through humble inquiry, using positive language with patients^19^ (eg, reinforcing their successes) and avoiding stigmatizing language notes in the electronic health record,^38,39^ with the goal of also promoting antiracism in the heads and hearts of clinicians. At a systems level, our findings suggest the role clinics and leadership may adopt in educating clinical teams about institutional racism. Clinics may also be able to implement antiracism health campaigns, similar to mass media campaigns to advocate for various diseases,^40^ and integrate health equity into quality of care metrics (such as racial disparities in health outcomes^41^), supporting recent calls for greater focus on health equity in quality improvement and health care implementation efforts.^26^ Importantly, participants noted the importance of compensation for these efforts such that they are truly integrated into the structure of the workplace in a sustainable manner. On an individual level, authentic positionality statement practices have been suggested in the context of health care research to encourage investigators to honestly acknowledge their position in relation to the work; our research suggests that these statements may be equally helpful in clinical care.^32^

Practices at the levels of individual clinician and health care system are fundamental to racial healing and start with education. Our work highlighted the importance of accurate training around the history of anti-Black racism in medicine; literature supports the urgency of this reckoning,^28,29^ prompting development of a comprehensive syllabus.^30^ Furthermore, in the setting of racially and ethnically charged police violence, our work emphasizes the need for more general educational sessions and trainings that promote racial justice. For example, we could find inspiration in Jones’ more recent work, building on levels of racism to identify mechanisms of racism through structures, policies, practices, and norms and values. In a global sense, we may be able to incorporate antiracism practices in medicine by being constantly on the lookout, embracing the discomfort that may be a barrier to culture change by consistently and intentionally asking, “How is racism operating here?” and being open to responses.^42^ More locally, deescalation trainings or bystander intervention training to interrupt microaggressions^43^ may help clinic staff avoid calling hospital police or security or disproportionately referring Black patients to medical regulatory bodies (such as child protective services) inappropriately.^31^

One cross-cutting theme that emerged from our analyses was synergy between levels of antiracism, with institutional antiracism discussed in conjunction with internalized and personally mediated antiracism. For example, one participant noted that showing up as her authentic self (internalized antiracism) was a foundation for dialogue with individuals (personally mediated antiracism), which in turn was a foundation for sharing ideas through formal trainings and workshops among colleagues (institutional antiracism). This synergy has health care implications; antiracism communication may be reinforced across levels. For instance, to promote mental health equity for Black patients, individual clinicians could check in with patients about mental health outcomes associated with racism, followed by referral to a collaborative care team comprising social work, primary care, psychology, and other team members.

### Feasibility of Health Care Adaptations

There may be several considerations for health care systems and individual clinicians to implement suggested health care adaptations of antiracism practices. For health care systems, implementing certain institutional antiracism practices, such as patient advocacy efforts and hiring staff to promote diversity, equity, and inclusion, will require significant time, money, and effort.^44^ For personally mediated antiracism practices, individual clinicians need to spend intentional time and mental effort on unlearning habits of racism, such as negative language in the health record, many of which are unfortunately unintentionally taught in medical education.^45^ There are institutional practices (eg, developing affiliate groups for staff) and personally mediated practices (eg, displaying signage aligned with racial justice) that may require fewer resources. Thus, the feasibility of suggested antiracism practices varies based on the type of practice; however, lasting change will require significant time, money, energy, and support.

This study has several strengths. The group of 40 interviewees had diverse racial and ethnic backgrounds, ages, and occupations; our findings reflect a wide range of experiences and settings. Study activities were conducted by a diverse and interdisciplinary research team with a variety of lived experiences, providing a unique lens for analysis, as well as adaptation to medicine.

### Limitations

This study has several limitations, and 3 major limitations should be noted. First, our interviewee pool may have biased our findings. Owing to recruitment logistics, many interviewees resided in California, decreasing geographical diversity. Second, we were unable to reach saturation within each field, and some occupations were not represented (eg, architecture), so findings may not reflect the full scope of strategies. Third, our initial intention and interview guide were skewed toward personally mediated practices. It is striking in this context that our interviews originated practices at levels of interpersonal and systemic racism; future exploration of those levels of racism may reveal many additional strategies because our data should not be seen as exhaustive in these domains.

## Conclusions

Applying transdisciplinary antiracism communication practices to medicine may be one mechanism to support transformative change that promotes health equity. Future implementation and evaluation of these antiracism practices should continue to use human-centered design approaches, employing a patient-centered approach to ensure that Black voices are lifted and heard. From individual clinicians to health care systems at large, medicine has the power and responsibility to make actionable change across all levels of antiracism.

## Notes

Supplement. **eAppendix.** Interview Guide **eTable 1.** Interview Team Demographics and Interview Logistics **eTable 2.** Codebook **eFigure.** 3 Emergent Antiracism Themes Mapped to 7 Codes
