# Supplementary material for: Nonmedical Transdisciplinary Perspectives of Black and Racially and Ethnically Diverse Individuals About Antiracism Practices: A Qualitative Study
Source: JAMA Netw Open. 2022 Feb 9;5(2):e2147835. doi: 10.1001/jamanetworkopen.2021.47835 (PMC8829657; doi:10.1001/jamanetworkopen.2021.47835)
Supplement: Supplement. — eAppendix. Interview Guide eTable 1. Interview Team Demographics and Interview Logistics eTable 2. Codebook eFigure. 3 Emergent Antiracism Themes Mapped to 7 Codes [file jamanetwopen-e2147835-s001.pdf]

Supplemental Online Content

Shankar M, Cox J, Baratta J, et al. Nonmedical transdisciplinary perspectives of Black and racially and ethnically diverse individuals about antiracism practices: a qualitative study. *JAMA Netw Open*. 2022;5(2):e2147835.
doi:10.1001/jamanetworkopen.2021.47835

eAppendix. Interview Guide
eTable 1. Interview Team Demographics and Interview Logistics
eTable 2. Codebook
eFigure. 3 Emergent Antiracism Themes Mapped to 7 Codes

This supplemental material has been provided by the authors to give readers additional information about their work.

© 2022 Shankar M et al. *JAMA Network Open.*

eAppendix. Interview Guide

*I am with Participant *ID*. Thank you for being willing to interview! We are conducting this to learn about anti-racist communication practices. We want to learn ways to promote equity for Black individuals, meaning those who fall under the social group from the African diaspora. We're going to be talking about concepts like racial justice, racism, racial trauma, and anti-racist practices. Are there any other terms you would rather we use to talk about this topic? We will follow your lead and use your language as much as possible. We are really looking to and hoping to learn from your experiences. Throughout the interview, feel free to pass on questions or stop at any time for any reason.*

<u>Appreciative Inquiry and Building Trust</u>

I'd love to start by learning about your job. What do you do?

Who are the people you work with? You might think about anyone you interact with, work with, or help in the work that you do, including clients, colleagues, students, secretaries, janitors. We'll talk about interactions with these people throughout the interview.

What do you do to connect with these people? What do you do to build trust with these people?

<u>Race in Interpersonal Interactions</u>

Now we're going to talk about how race might come up in the relationships you mentioned.

What are some examples of how race comes up in the interactions with the people you mentioned?

How has race contributed to work interactions in a positive way?

Can you describe some examples of conversations about race? When race does come up, what happens? If it is discussed, how? What are some examples of how you have approached these conversations? [prompts: implicit, subtle, unspoken vs explicit, overt, spoken]

<u>Experiences with Anti-Black Racism</u>

Now that we've talked about race a bit, I'd like to learn about times when anti-Black racist behaviors come up in the interactions we've been talking about. Again, feel free to pass on these questions or stop at any time for any reason. [Interviewer prepared to define anti-Black racism if necessary]

How have you witnessed overt or subtle actions of anti-Black racism? If so, can you expound a bit on this? (Probe: communication practices, specific words used, other interventions)

[To be asked only to Black participants] How have you witnessed or experienced overt or subtle actions of anti-Black racism? What has this looked like and how do you navigate it? (Probe: individual practices, specific communication methods, interventions, coping strategies)

<u>Anti-Racist Practices</u>

© 2022 Shankar M et al. *JAMA Network Open.*

What are some of the things you do to show support for Black individuals you interact with in your work?

What are some examples of behaviors you use to help Black individuals feel seen and heard when actions of anti-Black racism come up in the work that you do? [Again, thinking about anyone you work with including clients, colleagues, students, secretaries, janitors - really anyone part of your work/organization/institution].

What are some things that help to motivate you in speaking up against anti-Black racist behaviors in these interactions?

<u>Anti-Racism Workplace Needs</u>

How have you seen anti-Black racism being addressed in your work? Can you tell us a bit about this? You can reflect on appropriate vs inappropriate ways of addressing anti-Black racism.

How supported do you feel in your work environment in regard to efforts made to address anti-Black racism? If you feel supported, can you tell us some of the things you or your organization do that show this commitment?

What is your ideal for feeling supported and feeling like your work environment is addressing anti-Black racism?

What additional skills, training, education, etc would help in addressing anti-Black racism in the workplace?

<u>Reflection on Outcomes/Wrap-Up</u>

How do you know what you're doing is working or not working? Is there a way to tell that the behaviors that you participate in are (in)effective in addressing anti-Black racism?

Is there anything else that you would like to share or anything else that we should have asked?

© 2022 Shankar M et al. *JAMA Network Open.*

eTable 1. Interview Team Demographics and Interview Logistics

TEAM DEMOGRAPHICS		
	**Number (N = 10)**	**%**
**Race/Ethnicity**		
Black or African American	3	30%
Hispanic, Latinx, or Spanish origin	2	20%
East Asian, Southeast Asian, or South Asian	3	30%
White or European origin	2	20%
**Gender**		
Woman	9	90%
Non-Binary	1	10%
**Educational Level**		
Undergraduate student	2	20%
Bachelor's degree (public health, communications, biology, gender studies, pre-med)	3	30%
Master's degree (community health)	2	20%
Doctoral degree (PhD linguistics, PhD communication, MD internal medicine)	3	30%
**INTERVIEW LOGISTICS**		
	**Number (N = 40)**	**%**
**Interviewer/Interviewee**		
One-on-one	35	88%
Two-on-one	5	13%
**Interview Modality**		
Zoom video	32	80%
Zoom audio	7	18%
In-person	1	3%

© 2022 Shankar M et al. *JAMA Network Open.*

eTable 2. Codebook

Code	Description
Dialogue and humble inquiry	Conversation that builds connection, forges trust, as well as involves discussions about race, racism, and anti-racism that may also cause discomfort.
Building trust	The belief that someone will act in your best interest and arises from perceptions of the individual's values (including fidelity and honesty) and competence (Armstrong 2007).
Allyship/Shared humanity	Active, consistent, and arduous practice of unlearning and re-evaluating in which a person of privilege seeks to operate in solidarity with a marginalized group of people (Nixon 2019). Examples of non-Black people doing things to promote Black people, fill in the gaps of the work that needs to be done; this may not explicitly involve interactions with Black people, but allyship always involves actions that disrupt anti-Black racism in some way. Shared humanity, mutual respect, and bringing one's authentic and vulnerable self to interpersonal interactions as an anti-racist practice. These behaviors help in building trust (Kim 2017). At the same time, shared humanity can be used negatively to hide racism (Morton 2011). Each individual has a unique narrative that should be validated as a means towards racial justice (Chin 2010)
Education	Learning the true history of racism – and the importance of history re-written in the context of hidden racism, history in the field of work of a person (Bonam 2018)
Representation	Representation of Black individuals at the workplace, at all levels (Pitts 2005), including mentorship from people of similar backgrounds, whether in-person at the same organization or not, including innovative technologies of peer support (Showunmi 2006; Brunsma 2016)
Mentorship	Mentorship between individuals to promote anti-racism; counseling during racist experiences, mentorship from role-models, race concordant, or not.
Authenticity	Bringing one's authentic, vulnerable, and imperfect self to each interpersonal interaction, as a means of connection.

© 2022 Shankar M et al. *JAMA Network Open.*

eFigure. 3 Emergent Antiracism Themes Mapped to 7 Codes

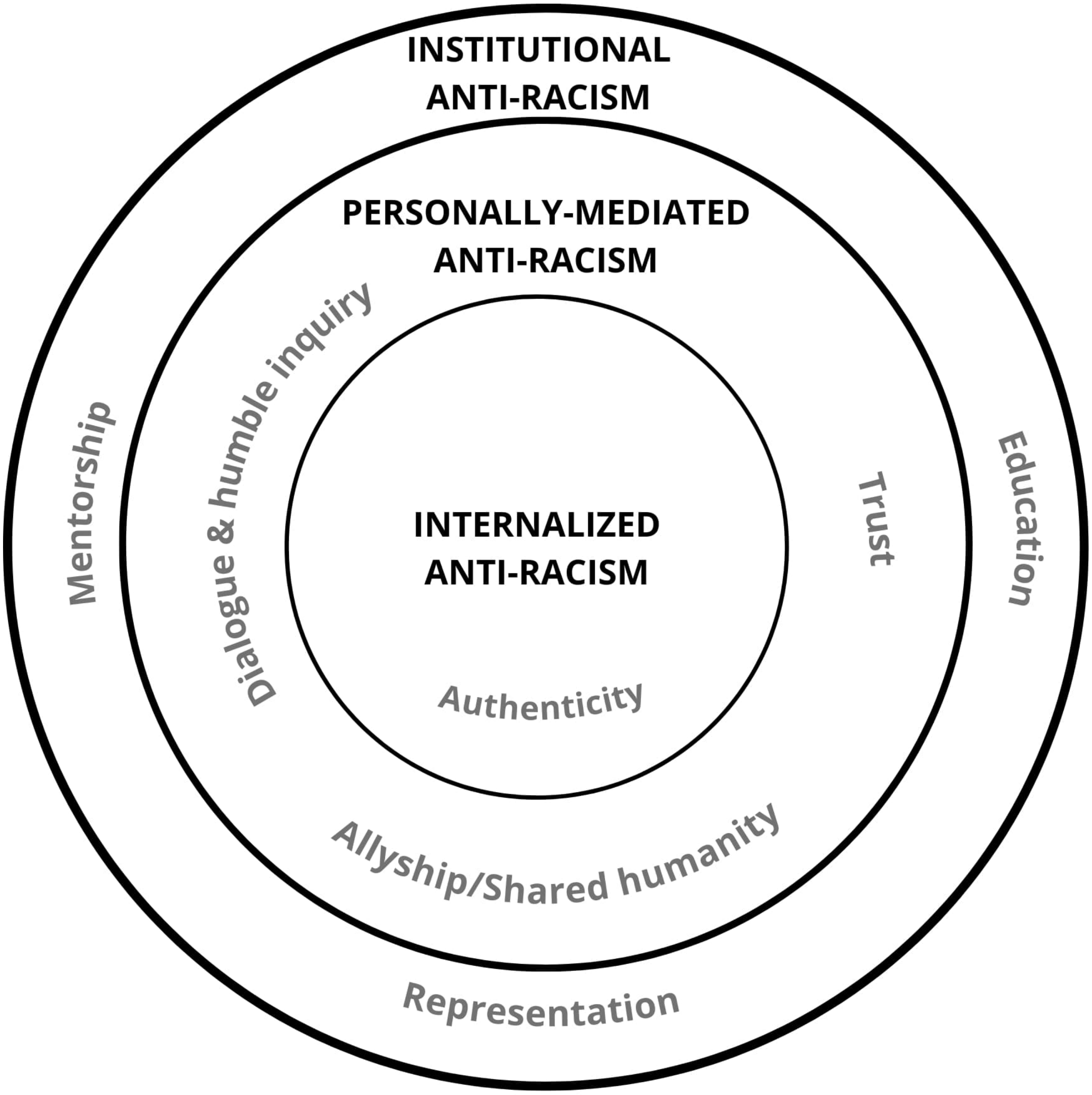

© 2022 Shankar M et al. *JAMA Network Open.*
